# Maxillary mesenchymal chondrosarcoma harboring *HEY1::NCOA2* fusion in a 13-year-old girl: a rare case report and literature review

**DOI:** 10.3389/fped.2026.1758538

**Published:** 2026-03-05

**Authors:** Şule Çalışkan Kamış, Begül Yağcı, Ayşe Selcan Koç, Güliz Durak, Ali Yitik

**Affiliations:** 1 Department of Pediatric Hematology and Oncology, Adana Faculty of Medicine, Adana City Training and Research Hospital, University of Health Sciences, Adana, Türkiye; 2 Department of Pediatric Radiology, Adana Faculty of Medicine, Adana City Training and Research Hospital, University of Health Sciences, Adana, Türkiye; 3 Department of Nuclear Medicine, Adana Faculty of Medicine, Adana City Training and Research Hospital, University of Health Sciences, Adana, Türkiye; 4 Department of Pathology, Adana Faculty of Medicine, Adana City Training and Research Hospital, University of Health Sciences, Adana, Türkiye

**Keywords:** HEY1::NCOA2 fusion, maxillary sinus tumor, mesenchymal chondrosarcoma, pediatric sarcoma, RNA sequencing, sirolimus, targeted therapy

## Abstract

**Background:**

Mesenchymal chondrosarcoma (MCS) is a rare and highly aggressive subtype of chondrosarcoma, accounting for less than 1% of all chondrosarcomas. It predominantly affects adolescents and young adults and frequently arises in craniofacial bones and soft tissues. Diagnosis is challenging because of significant histological overlap with other high-grade spindle cell sarcomas, particularly when the cartilaginous component is minimal or absent. The identification of the *HEY1::NCOA2* gene fusion has emerged as a highly specific molecular marker for MCS, substantially improving diagnostic accuracy and providing potential therapeutic implications.

**Case presentation:**

We report the case of a 13-year-old girl who presented with a 3-month history of progressive right cheek swelling. Imaging revealed a destructive mass in the right maxillary sinus. Histopathological evaluation demonstrated a high-grade spindle cell tumor, initially interpreted as fibrosarcoma, showing diffuse vimentin positivity, a high Ki-67 proliferation index (35%–40%), and CD34 negativity. Comprehensive molecular analysis confirmed a pathogenic *HEY1::NCOA2* gene fusion, while ETV6::NTRK3 fusion was excluded. The patient was treated with VAC chemotherapy (vincristine, actinomycin D, cyclophosphamide), local radiotherapy (60 Gy), cranial prophylactic radiotherapy (12 Gy), and subsequent debulking surgery. Follow-up ^18^F-FDG PET/CT demonstrated a partial metabolic response. Given persistent disease and molecular evidence suggesting activation of the PI3K/AKT/mTOR pathway in MCS, maintenance therapy with the mTOR inhibitor sirolimus was initiated.

**Conclusion:**

This case highlights the pivotal role of molecular diagnostics—particularly RNA sequencing—in establishing the diagnosis of mesenchymal chondrosarcoma and differentiating it from other high-grade pediatric sarcomas with overlapping morphology. Identification of the *HEY1::NCOA2* fusion not only confirms the diagnosis but may also support biologically targeted therapeutic strategies. Multimodal treatment incorporating chemotherapy, radiotherapy, surgery, and targeted maintenance therapy can achieve meaningful disease control in aggressive craniofacial MCS. To our knowledge, this represents one of the very few reported pediatric cases of maxillary MCS with confirmed *HEY1::NCOA2* fusion managed with sirolimus-based maintenance therapy.

## Introduction

Mesenchymal chondrosarcoma (MCS) is a highly aggressive and uncommon subtype of chondrosarcoma, accounting for less than 1% of all cases (1). It typically presents in adolescents and young adults and is characterized histologically by a distinctive biphasic pattern: small, undifferentiated round or spindle cells interspersed with islands of hyaline cartilage (1, 2). MCS can arise in both skeletal and extraskeletal sites, with a notable predilection for craniofacial bones, including the maxilla, mandible, orbit, and sinonasal region (2, 3).

Diagnosing MCS based solely on histopathology can be challenging, especially when the cartilaginous component is minimal or absent—situations in which the tumor may closely resemble other high-grade small round cell sarcomas, such as fibrosarcoma, Ewing sarcoma, or undifferentiated pleomorphic sarcoma (3, 4). This histologic overlap underscores the need for ancillary molecular techniques to improve diagnostic precision (4).

The identification of a recurrent *HEY1::NCOA2* gene fusion has represented a significant advance in the diagnostic workup of MCS. This fusion, resulting from an intrachromosomal deletion or a t(8;8) (q13;q21) translocation on chromosome 8, produces a chimeric transcription factor that combines the DNA-binding basic helix–loop–helix (bHLH) domain of HEY1 with the nuclear receptor coactivator domains of NCOA2 (2, 5). *HEY1::NCOA2* is detected in approximately 80%–90% of MCS cases and is rarely, if ever, present in other sarcoma subtypes, making it a highly specific molecular hallmark (1, 5).

The detection of this fusion can be achieved through a variety of molecular methods, including RNA sequencing, reverse transcription-polymerase chain reaction (RT-PCR), and fluorescence *in situ* hybridization (FISH) (5, 6). Among these, RNA sequencing offers the highest resolution and sensitivity for detecting rare or complex fusion variants but may be limited by cost and availability in resource-constrained settings. Functionally, the *HEY1::NCOA2* fusion has been implicated in oncogenesis by disrupting normal chondrogenic differentiation, altering Notch and RUNX2 signaling pathways, and promoting unregulated cellular proliferation (2, 7). Although it is considered diagnostically specific, the prognostic and therapeutic significance of different *HEY1::NCOA2* isoforms remains under investigation (7).

In the head and neck region, MCS accounts for up to 45% of cases and presents unique diagnostic and therapeutic challenges due to the complexity of regional anatomy and the potential for late local or distant recurrence (3, 8). While earlier detection in craniofacial sites may contribute to improved outcomes compared to other anatomical locations**,** long-term follow-up remains essential to monitor for relapse (3, 8, 9).

This report describes a rare case of maxillary MCS in a 13-year-old girl, confirmed to harbor the *HEY1::NCOA2* fusion through comprehensive RNA sequencing. Notably, this case is distinguished by the presence of three distinct fusion isoforms, an exceptionally rare molecular finding in the pediatric population. We outline her diagnostic evaluation, multimodal treatment approach, and early clinical course, highlighting the pivotal role of advanced molecular diagnostics in the management of rare pediatric sarcomas.

## Case presentation

A 13-year-old girl presented with a 3-month history of progressive right cheek swelling. Initial evaluation at a local dental faculty included imaging that revealed a lesion occupying the right maxillary sinus. An incisional biopsy performed on July 25, 2023, demonstrated a malignant mesenchymal tumor. On August 2, 2023, a fine-needle aspiration biopsy (FNAB) demonstrated a malignant mesenchymal tumor. Immunohistochemistry (IHC) showed weak Pan-TRK positivity with non-specific cytoplasmic staining, which was insufficient to support a diagnosis of TRK fusion-positive sarcoma.

**Figure 1:**
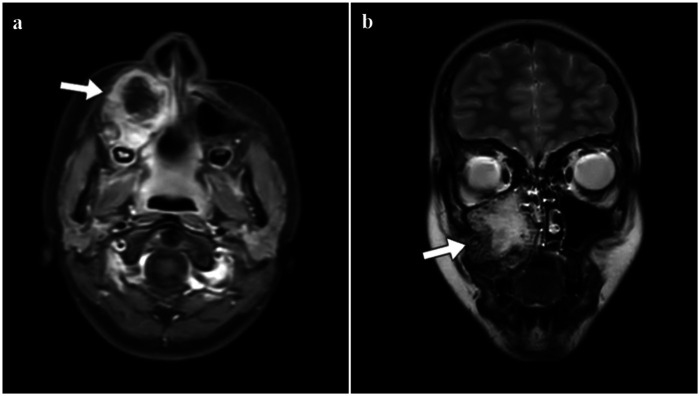
MRI of the face demonstrating a destructive maxillary lesion with soft tissue extension. **(a)** Axial T1-weighted contrast-enhanced MRI shows a heterogeneously enhancing mass in the right maxillary sinus (arrow), associated with erosion of the surrounding bony walls and invasion into adjacent soft tissues. **(b)** Coronal T1-weighted contrast-enhanced MRI demonstrates the extent of the lesion (arrow), further highlighting the destructive changes and soft tissue involvement.

MRI revealed a 40 × 35 mm heterogeneously enhancing lesion in the right maxillary sinus with destruction of the medial and inferior sinus walls and extension into surrounding soft tissues (Figure 1). The axial T1-weighted contrast-enhanced MRI demonstrated a heterogeneously enhancing mass occupying the right maxillary sinus, with erosion of the surrounding bony walls and invasion into adjacent soft tissues (Figure 1a). The coronal T1-weighted contrast-enhanced MRI further delineated the full extent of the lesion, emphasizing the destructive bone changes and soft tissue involvement (Figure 1b).

18F-FDG PET/CT performed on September 15, 2023, demonstrated a hypermetabolic lesion in the right maxilla (SUVmax: 6.2), as well as a second mild FDG-avid focus in the proximal left tibia (SUVmax: 3.7). This tibial finding was first identified at the time of initial staging and, in the absence of additional radiologic or clinical features suggestive of malignancy and without further imaging beyond PET/CT, was interpreted as a benign fibrous cortical defect.

Histopathological examination of the initial maxillary biopsy performed on July 25, 2023, revealed monomorphic spindle-shaped tumor cells with hyperchromatic nuclei and eosinophilic cytoplasm. No cartilage islands were identified. IHC demonstrated diffuse vimentin positivity, while CD34, SMA, S100, desmin, myogenin, and SS18 immunostaining were negative, and the Ki-67 proliferation index was approximately 35%–40%. These findings were consistent with a malignant mesenchymal tumor favoring fibrosarcoma, and molecular testing was recommended to exclude ETV6::NTRK3 gene fusion.

### Histopathological findings

Initial biopsy (July 25, 2023): Microscopic examination of the incisional biopsy revealed fragments of a hypercellular malignant mesenchymal neoplasm composed predominantly of monomorphic spindle-shaped tumor cells arranged in short fascicles. The tumor cells showed elongated, hyperchromatic nuclei, inconspicuous nucleoli, and moderate amounts of eosinophilic cytoplasm, with readily identifiable mitotic figures. No hyaline cartilage islands, osteoid production, or definite biphasic architecture were identified in the sampled tissue. Immunohistochemistry demonstrated diffuse cytoplasmic positivity for vimentin (Table 1), while CD34, SMA, S100, desmin, myogenin, and SS18 immunostaining were negative; the Ki-67 proliferation index was approximately 35%–40% (Table 1). Overall, the findings were consistent with a high-grade spindle cell sarcoma, and molecular testing was recommended to exclude an ETV6::NTRK3 fusion.

**Table 1:** Immunohistochemical findings in all tumor specimens.

Antibody	Initial Biopsy (July 25, 2023)	Repeat Biopsy (August 2, 2023)	Resection Specimen (May 20, 2025)	Interpretation
Vimentin	Diffuse positive	Diffuse positive	Diffuse positive	Consistent with mesenchymal origin
Pan-TRK	Not performed	Weak, non-specific cytoplasmic	Negative	Insufficient for TRK fusion-positive sarcoma
CD34	Negative	Negative	Negative	Against solitary fibrous tumor
SMA	Negative	Negative	Negative	Against smooth muscle differentiation
S100	Negative	Negative	Negative	Against neural or cartilaginous lineage
Desmin	Negative	Negative	Negative	Against myogenic tumor
Myogenin	Negative	Negative	Negative	Against rhabdomyosarcoma
SS18 (IHC)	Negative	Negative	Negative	Against synovial sarcoma
CD99	Not performed	Not performed	Focal positive	Non-specific
Ki-67	∼35%–40%	High	High	High proliferative activity

IHC, immunohistochemistry; Pan-TRK, pan-tropomyosin receptor kinase; SMA, smooth muscle actin. Ki-67, proliferation index is expressed as the approximate percentage of positively stained tumor cell nuclei. Weak Pan-TRK staining was interpreted as non-specific cytoplasmic reactivity and was insufficient to support a diagnosis of TRK fusion–positive sarcoma.

Repeat biopsy (August 2, 2023): The repeat biopsy again demonstrated a malignant spindle cell tumor, with focal transition toward round-cell areas and increased nuclear pleomorphism and mitotic activity. The growth pattern remained largely patternless and lacked a well-formed biphasic configuration, and definitive hyaline cartilage islands were not identified. Weak, non-specific cytoplasmic Pan-TRK staining was observed and was considered insufficient to support a TRK fusion–positive sarcoma (Table 1). Given the persistent diagnostic ambiguity, advanced molecular analysis was recommended for definitive diagnostic clarification.

**Figure 2:**
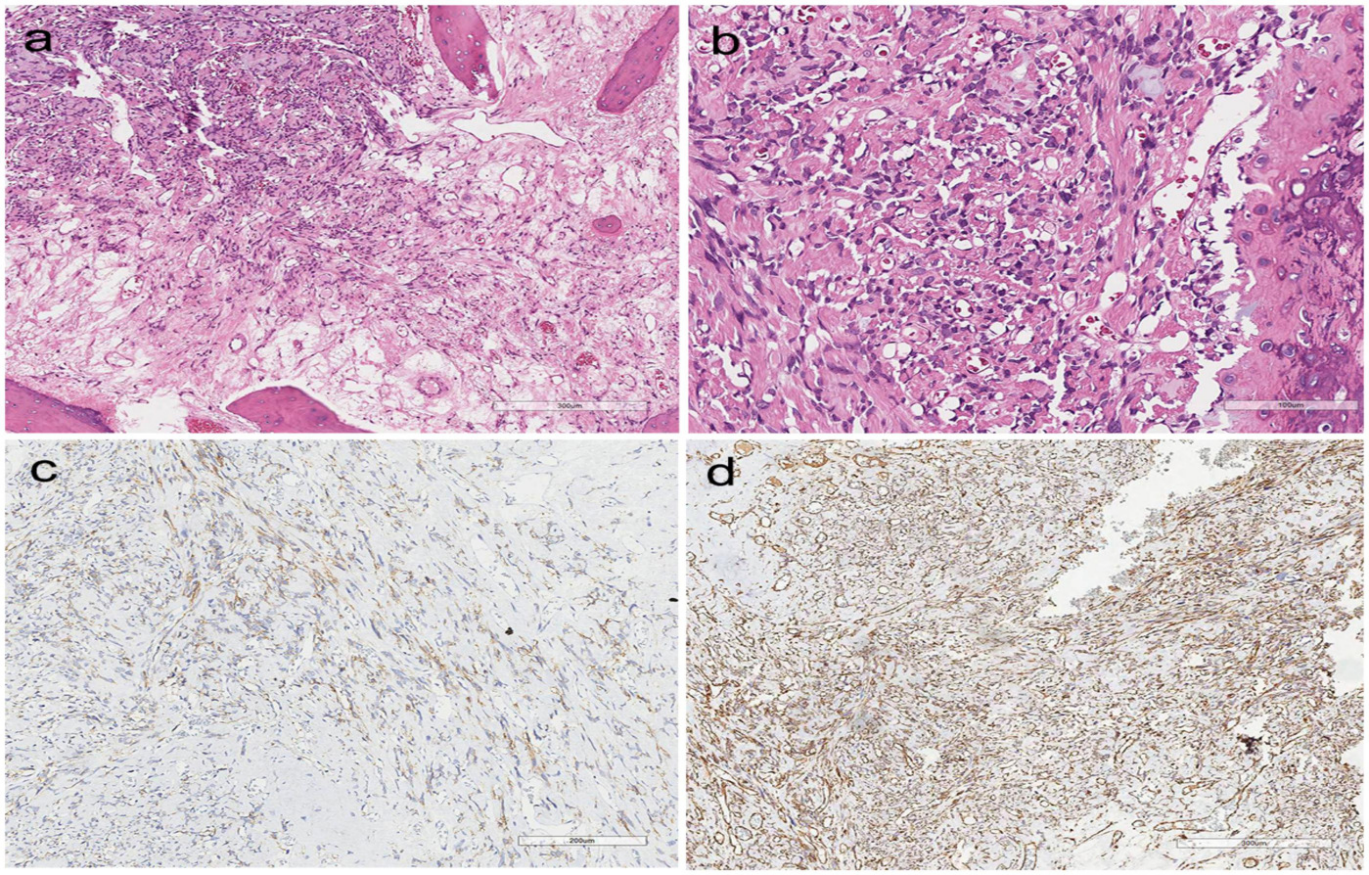
Histopathological findings of the resection specimen. **(a)** H&E, ×200: hypercellular areas composed of spindle-shaped tumor cells with marked nuclear atypia. **(b)** H&E, ×400: high-power view demonstrating pleomorphic nuclei and increased mitotic activity. **(c)** CD99 immunostaining, ×200: focal cytoplasmic positivity. **(d)** vimentin immunostaining, ×200: diffuse cytoplasmic positivity in tumor cells.

Definitive resection specimen (May 20, 2025): Histopathological evaluation of the Caldwell–Luc resection specimen demonstrated a high-grade malignant mesenchymal tumor characterized by hypercellular areas of spindle-shaped tumor cells with marked nuclear atypia (Figure 2a). High-power examination showed pleomorphic nuclei and brisk mitotic activity (Figure 2b). Although focal areas suggestive of chondroid differentiation were identified, the classic biphasic pattern of mesenchymal chondrosarcoma was largely absent. Immunohistochemistry on the resection specimen showed focal CD99 cytoplasmic positivity (Figure 2c; Table 1) and diffuse vimentin positivity (Figure 2d; Table 1). Taken together, the atypical morphology and limited cartilaginous differentiation underscored the critical role of molecular profiling for definitive classification.

Representative histopathological and immunohistochemical features of the resection specimen are shown in Figure 2, and the immunohistochemical findings from all biopsy and resection specimens are summarized in Table 1.

**Figure 3:**
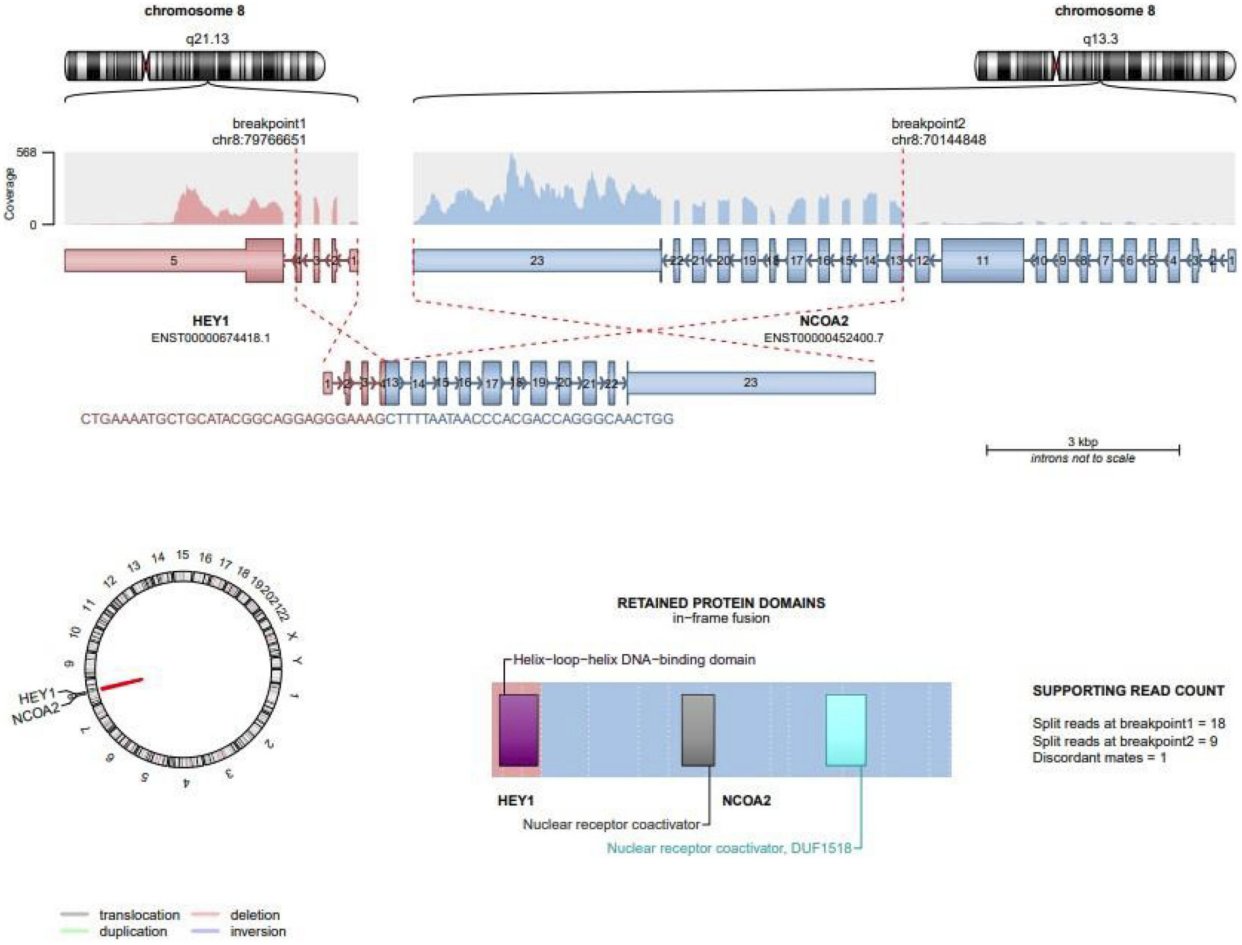
Schematic representation of HEY1::NCOA2 fusion. Schematic illustration of the canonical HEY1::NCOA2 gene fusion detected in the patient's tumor. The chromosomal rearrangement involves breakpoints at 8q13.3 and 8q21.13, fusing HEY1 exons 1–4 to NCOA2 exons 13–23. The resulting chimeric transcript retains the basic helix–loop–helix (bHLH) domain of HEY1 and the nuclear receptor coactivator domain of NCOA2. Supporting RNA-Seq read counts confirm this pathogenic event.

To clarify the diagnosis, comprehensive transcriptome RNA sequencing was performed. It identified a pathogenic *HEY1::NCOA2* gene fusion, with HEY1 exons 1–4 joined to NCOA2 exons 13–23. Three pathogenic deletions in chromosome 8q13.3–q21.13 were detected, indicating genomic instability in the region harboring the HEY1 and NCOA2 genes and supporting the presence of two subclonal fusion transcripts. Fusion transcripts were detected at the RNA level, confirming transcriptionally active rearrangements rather than protein-level findings. This fusion is considered a highly specific molecular hallmark for mesenchymal chondrosarcoma (MCS), reported in approximately 80%–90% of cases in recent studies. The schematic representation of this canonical *HEY1::NCOA2* fusion is shown in Figure 3.

**Figure 4:**
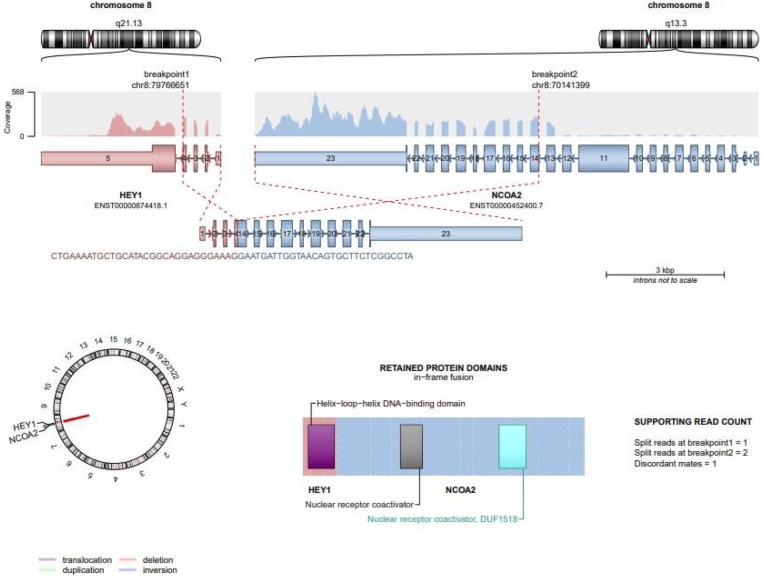
Alternative in-frame fusion isoform of HEY1::NCOA2. A second HEY1::NCOA2 transcript was identified involving fusion of HEY1 exon 5 with NCOA2 exons 11–23. Despite a different breakpoint, this in-frame variant also retains critical functional domains, including the bHLH domain of HEY1 and the coactivator domains of NCOA2. Read support from split reads and discordant mate-pairs validated the presence of this transcript.

Further RNA-seq analysis revealed a second in-frame fusion isoform, involving HEY1 exon 5 and NCOA2 exons 11–23. This alternative transcript also retained critical functional domains and was validated by split reads and discordant mate-pairs (Figure 4).

**Figure 5:**
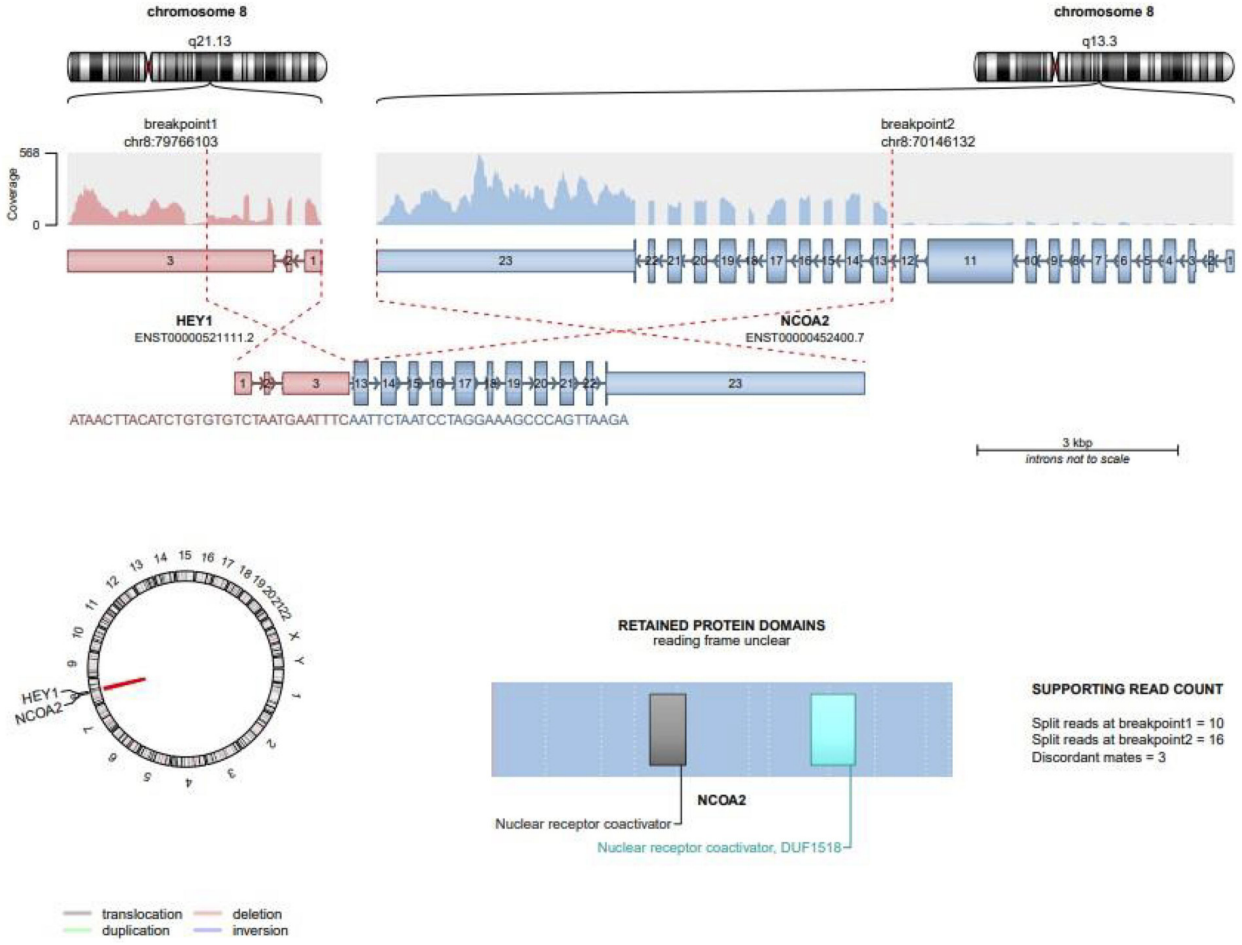
Third variant of HEY1::NCOA2 fusion. A third, rarer fusion variant was detected involving HEY1 exon 3 and NCOA2 exon 11. Unlike the canonical and alternative in-frame fusions, this transcript has an undefined open Reading frame. However, RNA sequencing confirmed its transcriptional presence. Retained domains include portions of the NCOA2 nuclear receptor coactivator region.

A third fusion variant was also detected, involving HEY1 exon 3 and NCOA2 exon 11. Although this transcript had an undefined open reading frame, its transcriptional presence was confirmed. It retained portions of the NCOA2 nuclear receptor coactivator domain (Figure 5).

Following confirmation of the diagnosis, the patient was diagnosed with mesenchymal chondrosarcoma of the maxilla. She received VAC chemotherapy and local radiotherapy (60 Gy in 33 fractions) between May and June 2024. Additionally, cranial prophylactic RT (12 Gy in 8 fractions) was administered.

**Figure 6:**
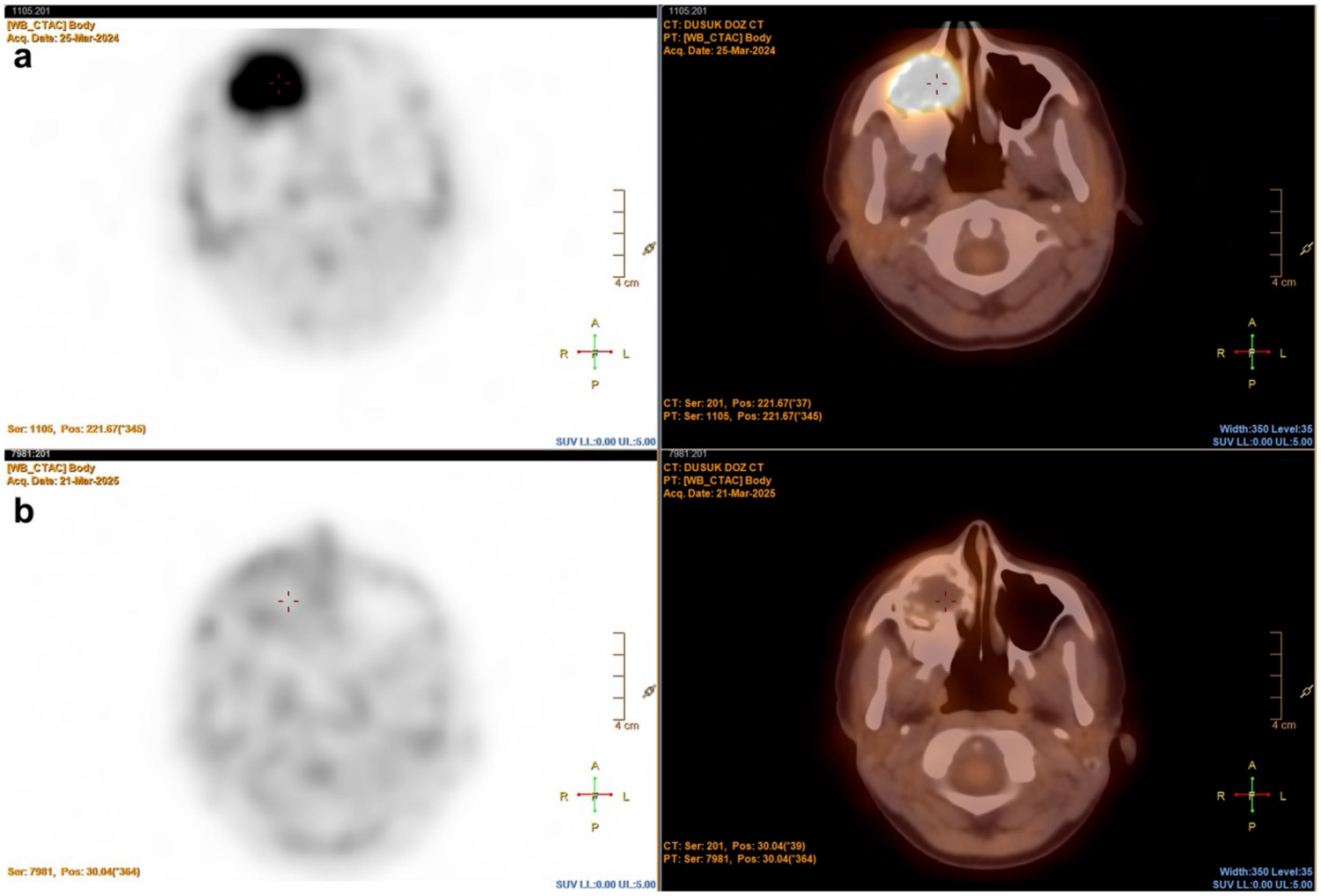
Comparative ^18^F-FDG PET/CT images before and after treatment. (a) Baseline ^18^F-FDG PET/CT scan (acquired on March 25, 2024) demonstrating a hypermetabolic lesion in the right maxillary sinus (SUVmax = 6.2), consistent with metabolically active high-grade sarcoma. (b) Post-treatment ^18^F-FDG PET/CT scan (acquired on March 21, 2025) showing complete metabolic response with no residual FDG uptake at the lesion site.

Follow-up 18F-FDG PET/CT in August 2024 demonstrated a partial metabolic response in the primary tumor (SUVmax: 4.08) (Figure 6a).

The tibial lesion showed no FDG uptake and was radiologically consistent with a benign fibrous cortical defect, emphasizing the importance of careful interpretation of incidental PET findings in pediatric oncology patients.

Debulking surgery was performed on May 20, 2025, and histopathological evaluation of the surgical specimen confirmed residual mesenchymal chondrosarcoma. Based on literature evidence indicating dysregulation of the mTOR signaling pathway in mesenchymal tumors (10), sirolimus was initiated as maintenance therapy (target dose: 1–2 mg/m^2^/day). A subsequent ^18^F-FDG PET/CT performed in March 2025 demonstrated complete metabolic response with no residual FDG uptake at the primary lesion site (Figure 6b).

As of the most recent follow-up in October 2025, the patient remained clinically and radiologically stable, with no evidence of new metastases and sustained disease control in the maxillary region. Comparative MRI assessment demonstrated necrosis of the osseous lesion in the right maxillary sinus with mild interval regression, findings that further support a radiologic response to sirolimus therapy.

## Discussion

Maxillary localization of tumors initially interpreted as fibrosarcoma is exceedingly rare in the pediatric population and requires thorough histopathologic and molecular characterization to exclude more common or targetable spindle cell neoplasms. The differential diagnosis includes other high-grade spindle cell tumors such as alveolar and embryonal rhabdomyosarcoma, NTRK-rearranged spindle cell sarcoma, and undifferentiated pleomorphic sarcoma (11). Given the histologic overlap, especially in tumors with minimal or absent cartilaginous components, mesenchymal chondrosarcoma (MCS) may initially be misdiagnosed as fibrosarcoma or other small round cell tumors (12).

Immunohistochemistry aids in narrowing the differential but often lacks specificity. In our case, diffuse vimentin positivity supported mesenchymal origin but was nonspecific, while the absence of CD34 and high Ki-67 proliferation index (>35%) indicated an aggressive phenotype. Importantly, *ETV6::NTRK3* fusion testing was negative, helping to rule out infantile fibrosarcoma—a TRK-fusion-positive entity with excellent response to TRK inhibitors such as larotrectinib and entrectinib (13, 14).

*HEY1::NCOA2* gene fusion has been reported in approximately 80%–90% of histologically confirmed mesenchymal chondrosarcomas (MCS) (15, 16). Nevertheless, a minor subset of MCS cases lacking this fusion has been described, with alternative genetic alterations such as *IRF2BP2::CDX1* and other rare rearrangements reported in the literature (15). Conversely, *HEY1::NCOA2* fusion has been only exceptionally identified in other sarcoma subtypes, underscoring its high—though not absolute—specificity for MCS within an appropriate histopathological context (16).

In the present case, the definitive diagnosis was established by comprehensive RNA sequencing, which identified a pathogenic *HEY1::NCOA2* fusion, thereby resolving the diagnostic ambiguity created by the atypical histomorphology. Beyond its diagnostic utility, this fusion is implicated in MCS oncogenesis through dysregulation of Notch signaling pathways and impaired chondrogenic differentiation, providing important biological insight into tumor development (17).

To date, the simultaneous presence of three distinct *HEY1::NCOA2* fusion transcript variants within a single tumor has not been previously reported in mesenchymal chondrosarcoma or other sarcoma subtypes, to the best of our knowledge. This finding may reflect intratumoral heterogeneity and subclonal genomic complexity.

The incidental detection of an FDG-avid lesion in the tibia raised concern for metastatic spread; however, subsequent imaging and metabolic quiescence suggested a benign fibrous cortical defect. Such false positives on PET/CT imaging are not uncommon in pediatrics and highlight the need for multidisciplinary interpretation of imaging findings in conjunction with clinical and histopathologic data (18).

Treatment of MCS often includes multi-agent chemotherapy regimens extrapolated from protocols for Ewing sarcoma and soft tissue sarcomas, as prospective data specific to MCS are lacking due to its rarity. VAC chemotherapy (vincristine, actinomycin D, cyclophosphamide) has demonstrated utility, particularly in the neoadjuvant setting when complete surgical resection is difficult due to anatomical constraints (19). In craniofacial MCS, radiotherapy is often necessary for local control, with recent studies supporting the use of proton therapy or high-dose conformal RT to minimize toxicity while achieving durable response (20).

Furthermore, recent evidence suggests that the PI3K/AKT/mTOR signaling pathway may be aberrantly activated in MCS, providing a rationale for incorporating mTOR inhibitors such as sirolimus in the maintenance setting, especially in patients with residual disease or poor surgical candidates (21, 22). This biologically targeted approach may offer prolonged disease control and improved quality of life, although prospective data are still limited.

### Differential diagnosis

The differential diagnosis of a high-grade spindle to round cell tumor arising in the maxilla of a pediatric patient includes Ewing sarcoma, small cell osteosarcoma, synovial sarcoma, fibrosarcoma, and malignant peripheral nerve sheath tumor (MPNST).

Ewing sarcoma may show sheets of uniform small round cells and diffuse CD99 positivity; however, the absence of diffuse membranous CD99 staining and the lack of *EWSR1::FLI1* or related fusions excluded this diagnosis.
Small cell osteosarcoma is characterized by malignant osteoid production, which was not identified in any specimen.
Synovial sarcoma was excluded based on negative SS18 immunostaining and the absence of *SS18::SSX* fusion.
Fibrosarcoma was initially favored due to the spindle cell morphology and diffuse vimentin positivity; however, fibrosarcomas lack recurrent gene fusions such as *HEY1::NCOA2*.
MPNST typically shows S100 positivity in at least a subset of cases, which was absent.

The identification of a pathogenic *HEY1::NCOA2* fusion ultimately supported the diagnosis of mesenchymal chondrosarcoma, despite the atypical histomorphology.

In summary, this case underscores the importance of integrated diagnostics—including advanced molecular techniques—in establishing the correct diagnosis of rare pediatric head and neck sarcomas. Multimodal therapy combining surgery, chemotherapy, radiotherapy, and molecularly targeted maintenance therapy may improve outcomes in patients with aggressive or unresectable MCS.

## Conclusion

This case underscores the complexities in diagnosing and managing rare pediatric mesenchymal chondrosarcomas of the maxillofacial region. Comprehensive evaluation integrating advanced imaging, detailed histopathological assessment, and molecular diagnostics is critical for accurate classification. A multidisciplinary management approach enables timely treatment planning and may improve outcomes in these rare and aggressive pediatric malignancies.

## Data Availability

The original contributions presented in the study are included in the article/Supplementary Material, further inquiries can be directed to the corresponding author.
